# Transcriptomics of the Interaction between the Monopartite Phloem-Limited Geminivirus Tomato Yellow Leaf Curl Sardinia Virus and *Solanum lycopersicum* Highlights a Role for Plant Hormones, Autophagy and Plant Immune System Fine Tuning during Infection

**DOI:** 10.1371/journal.pone.0089951

**Published:** 2014-02-28

**Authors:** Laura Miozzi, Chiara Napoli, Luca Sardo, Gian Paolo Accotto

**Affiliations:** 1 Istituto di Virologia Vegetale, (National Research Council) CNR, Torino, Italy; 2 Viral Recombination Section, HIV Drug Resistance Program, Center for Cancer Research, National Cancer Institute, Frederick, Maryland, United States of America; University of Turin, Italy

## Abstract

Tomato yellow leaf curl Sardinia virus (TYLCSV), a DNA virus belonging to the genus *Begomovirus*, causes severe losses in tomato crops. It infects only a limited number of cells in the vascular tissues, making difficult to detect changes in host gene expression linked to its presence. Here we present the first microarray study of transcriptional changes induced by the phloem-limited geminivirus TYLCSV infecting tomato, its natural host. The analysis was performed on the midrib of mature leaves, a material naturally enriched in vascular tissues. A total of 2206 genes were up-regulated and 1398 were down-regulated in infected plants, with an overrepresentation of genes involved in hormone metabolism and responses, nucleic acid metabolism, regulation of transcription, ubiquitin-proteasome pathway and autophagy among those up-regulated, and in primary and secondary metabolism, phosphorylation, transcription and methylation-dependent chromatin silencing among those down-regulated. Our analysis showed a series of responses, such as the induction of GA- and ABA-responsive genes, the activation of the autophagic process and the fine tuning of the plant immune system, observed only in TYLCSV-tomato compatible interaction so far. On the other hand, comparisons with transcriptional changes observed in other geminivirus-plant interactions highlighted common host responses consisting in the deregulation of biotic stress responsive genes, key enzymes in the ethylene biosynthesis and methylation cycle, components of the ubiquitin proteasome system and DNA polymerases II. The involvement of conserved miRNAs and of solanaceous- and tomato-specific miRNAs in geminivirus infection, investigated by integrating differential gene expression data with miRNA targeting data, is discussed.

## Introduction

Geminiviruses are a large family of plant viruses able to infect a wide variety of plants worldwide. Virions have a geminate shape of about 18×30 nm in size. The family is currently divided in seven genera, *Becurtovirus*, *Begomovirus*, *Curtovirus*, *Eragrovirus*, *Mastrevirus*,*Topocuvirus* and *Turncurtovirus* according to their genome organization, insect vector and host range [1], [2]. All viruses belonging to these genera have a single-stranded circular DNA (ssDNA) genome and replicate in the nuclei of the host cells. The genus *Begomovirus*, with its more than 200 species, is the most represented. It includes viruses with either monopartite or bipartite genomes, transmitted by the whitefly *Bemisia tabaci* Genn., infecting a wide range of dicots in both the Old and the New World [3].

The begomovirus *Tomato yellow leaf curl Sardinia virus* (TYLCSV), as well as other related viruses, is responsible for the tomato yellow leaf curl disease (TYLCD), that in the last decades has devastated tomato (*Solanum lycopersicum* L.) crops in all tropical and sub-tropical regions [4], [5]. Typical symptoms are growth stunting, curling of leaf margins, yellowing of young leaves and floral abortion.

TYLCSV is a phloem-limited geminivirus, with a monopartite genome containing six open reading frames, bidirectionally organized in two transcriptional units coding for a coat protein (CP), two proteins related to replication (Rep and Ren), a movement protein (V2), a transcription activator protein (TrAP) and a pathogenicity factor C4 [6]. Several interactions between geminiviral proteins and host factors [7], able to impact on plant gene expression, are known. For example, Rep interacts with the host retinoblastoma-related protein (RBR),which represses the cell cycle progression by its interaction with E2F [8], [9]. The TrAP protein (also known as AL2 or C2) inactivates the adenosine kinase (ADK), thus intervening on transcriptional gene silencing (TGS) [10]. This viral protein also interacts with the COP9 signalosome, altering the cellular processes regulated by the SCF (Skp, Cullin, F-box) complexes, including the jasmonate signalling, and regulating host response to infection [11].

Plant-virus interaction is a complex system and the ability of a virus to effectively infect its host depends on the accurate balance between host defences and virus counteracting strategies. Up to now, several studies have been performed to characterize the transcriptional responses of host plants to RNA viruses [12], [13], [14], [15], [16], but only a few dealt with the global impact of geminiviruses on host transcriptome. Naqvi and coworkers [17], by using a subtractive hybridization approach, identified 20 differentially expressed sequence tags (ESTs) induced by the bipartite *Tomato yellow leaf curl New Delhi virus* (ToLCNDV) in tomato. Góngora-Castillo and co-workers [18] used mRNA-seq technology to compare the transcriptome of recovered and not-recovered pepper leaves infected by the bipartite phloem-limited *Pepper golden mosaic virus* (PepGMV). Up to now, only two studies used a microarray approach to analyse the transcriptional changes of host plant in response to geminiviral infection. In the first one, Ascencio-Ibanez and colleagues [19] found that the infection of the bipartite begomovirus *Cabbage leaf curl virus* (CabLCV) alters the expression of several genes related to cell cycle in the model plant Arabidopsis. In the second one, Pierce and Rey [20], analysed the bipartite begomovirus *South African cassava mosaic virus* (SACMV)/Arabidopsis interaction by looking at the time course of the infection, from early to late symptomatic stages. Both these studies concerned begomoviruses that are able to escape the phloem and spread in the surrounding tissues, and used an experimental host plant. We decided to perform a microarray analysis of transcriptomic changes induced by a phloem-limited begomovirus in its natural host [4], using tomato infected by TYLCSV.

Tomato is currently considered a model species in the Solanaceae family, with complete genome sequence recently released [21] and several dedicated genetic and genomic resources available (http://solgenomics.net/). Moreover, it is considered one of the most economically relevant crops worldwide and is a well known natural host of TYLCSV. Taken together, these aspects prompted us to select the tomato-TYLCSV compatible interaction for our analysis. To our knowledge, our research represents the first microarray study of the transcriptional responses induced by a phloem-limited monopartite geminivirus in its natural host plant.

## Materials and Methods

### Biological materials

*Solanum lycopersicum* (cv Moneymaker) plants were either infected using agroinoculation with LBA4404 cells carrying an infectious clone of TYLCSV (Acc. no. X61153, [22]) or mock-inoculated with an empty pBIN19 plasmid. Plants were maintained in a growth chamber at 24°C with a photoperiod of 14 h light/10 h dark. Central portions of the midrib of mature (fifth) leaf were harvested from infected and mock-inoculated plants six weeks post inoculation (wpi), a timing selected to allow a full symptom expression. For each condition, a total of 20 plants were considered and pooled in four biological replicates (four, five plants per pool).

### Southern blot analysis

Viral replication was examined by Southern blot analysis. Total DNA was extracted with TLES buffer (5% sodium dodecyl sulfate [SDS] 150 mM LiCl, 50 mM Tris-HCl, 5 mM EDTA [pH 9.0]). After electrophoresis in 1% agarose gels containing 0.5 µg of ethidium bromide per ml in 0.5× Tris-borate-EDTA, nucleic acids were blotted onto positively-charged nylon membranes (Boehringer Mannheim). A digoxigenin-labelled TYLCSV-specific probe was used for hybridization at 65°C in a standard hybridization mix (Boehringer), followed by high-stringency washes. CDP-Star (Tropix) was used for chemiluminescent detection.

### RNA extraction and microarray experiment

Total RNA was extracted with Trizol reagent (Ambion) following the manufacturer's instructions. RNA concentration and purity were determined with the NanoDrop ND-1000 spectrophotometer (Thermo Scientific, Wilmington, USA). RNA integrity was examined with the Experion Automated Electrophoresis Station (Bio-Rad). cDNA synthesis, hybridization, washing and scanning were performed by IMGM Laboratories (www.imgm.com) as follows: 500 ng of total RNA were spiked with *in vitro* synthesized polyadenylated transcripts (One-Color RNA Spike-In Mix, Agilent Technologies), which serve as internal labelling control for linearity, sensitivity and accuracy. The spiked total RNA was reverse transcribed into cDNA and then converted into labelled cRNA by *in vitro* transcription (Quick-Amp Labeling Kit One-Color, Agilent Technologies) incorporating Cyanine-3-CTP. cRNA concentration (ng/µl), RNA absorbance ratio (260 nm/280 nm) and Cyanine-3 dye concentration (pmol/µl) were checked for all cRNA samples using the NanoDrop ND-1000 UV-VIS spectrophotometer. The RNA 6000 Nano LabChip Kit (Agilent Technologies) was used on the 2100 Bioanalyzer (Agilent Technologies) to analyze the quality of labelled non-fragmented cRNA. Following cRNAclean-up and quantification (NanoDrop ND-1000), 1.65 µg of each Cyanine-3-labeled cRNA sample was fragmented and prepared for One-Color based hybridization (Gene Expression Hybridization Kit, Agilent Technologies). cRNA samples were hybridized at 65°C for 17 h on separate Tomato Gene Expression Microarrays 4×44K (Agilent Technology) containing 43803 probes, corresponding to about 33000 tomato genes (expressed sequences). Fluorescent signal intensities were detected with Scan Control 8.4.1 Software (Agilent Technologies) on the Agilent DNA Microarray Scanner and extracted from the images using Feature Extraction 10.5.1.1 Software (Agilent Technologies). The data were sent back to our laboratory for further analyses. Normalization and analysis of microarray data were carried out using the Limma package [23]. Within and between array normalization was performed (Lowess normalization). Genes having a false discovery rate <0.05 were considered differentially expressed. Data have been submitted to ArrayExpress (www.ebi.ac.uk/arrayexpress; accession no. E-MEXP-3953).

### qRT-PCR analysis

RNA samples were treated with Turbo DNase free (Ambion, Foster City, CA, U.S.A.) according to the manufacturer's instructions. DNA absence was then evaluated by RT-PCR using 18S rRNA-specific primers and the One Step RT-PCR kit (Qiagen). Single-strand cDNA was obtained from approximately 1,500 ng of total RNA using Oligo-dT (Invitrogen) primers and StrataScript reverse transcriptase (Stratagene, La Jolla, CA, U.S.A.). Volume of RNA samples was brought to 40 µl and then 10 µl of a mix composed of 0.6 µl of Oligo-dT at 500 ng/µl and 9.4 µl of distilled water was added. Samples were incubated for 5 min at 65°C and 10 min at room temperature. A master mix (8.5 µl) containing 5 µl of StrataScript RT buffer, 1 µl of RNase inhibitor (40 U/µl), 2 µl of dNTPs (10 mM), and 0.5 µl of RT StrataScript enzyme was then added and samples were incubated at 42°C for 1 h.

Quantitative PCR assays were carried out in a Step One Plus Real-Time PCR System (Applied Biosystems). Reactions were conducted in a total volume of 10 µl, containing 5 µl of Platinum Sybr Green qPCR Supermix-UDG (Invitrogen), 300 nM of each primer (Table S1), and 20 ng of cDNA template. The PCR cycling program consisted of: 50°C for 3 min, 95°C for 3 min, and 40 cycles each consisting of 95°C for 30 s and 60°C for 30 s. A melting curve (55 to 95°C with a heating rate of 0.5°C for 10 s and a continuous fluorescence measurement) was recorded at the end of each run to assess amplification product specificity. All reactions were performed with three technical and three biological replicates. PCR efficiency was determined from standard curves obtained using serial dilutions of tomato genomic DNA. The comparative threshold cycle method [24] was used to calculate the relative expression level using the tomato ubiquitin-conjugating enzyme *ubc* (GenBank Acc. No. AK324262.1) as reference gene.

### Functional analysis

Gene Ontology (http://www.geneontology.org/) annotation was obtained using Blast2go software [25], with default parameters. Lists of up- or down-regulated genes were searched for overrepresented GO terms. P values were computed with Fisher's exact test and a P value <10^−3^ was considered statistically significant [26]. The analysis was performed using a set of Perl and C programs available from the authors upon request.

## Results

### Experimental design

To guarantee a fully developed infection, sampling was performed 6 weeks after TYLCSV inoculation, when systemic symptoms such as leaf curling, yellowing and growth reduction, were evident.

In plant apexes and young leaves, DNA replication as well as cell division and expansion, may obscure the transcriptional changes due to virus infection. Based on this consideration, we hypothesized that viral effects on host gene expression would be more easily detectable in mature leaves, where developmental effects related to DNA replication and cell division are minimal. Therefore, we selected completely developed mature leaves to perform the microarray analysis.

It is also reasonable to assume that the plant transcriptional responses to the viral infection are, at least in part, specific to the cells/tissues where the pathogen is confined. TYLCSV, like other geminiviruses, is phloem-limited and i*n situ* hybridization studies indicate that its accumulation in tomato is mainly restricted to the nuclei of phloem and companion cells, with a number of infected cells in the order of 1∶100 [27]. To obtain biological samples enriched in infected cells and to maximize the viral transcriptional impact, we selected for the analysis the central portion of the midrib of a mature (fifth) leaf which is naturally enriched in phloem tissue.

**Figure 1:**
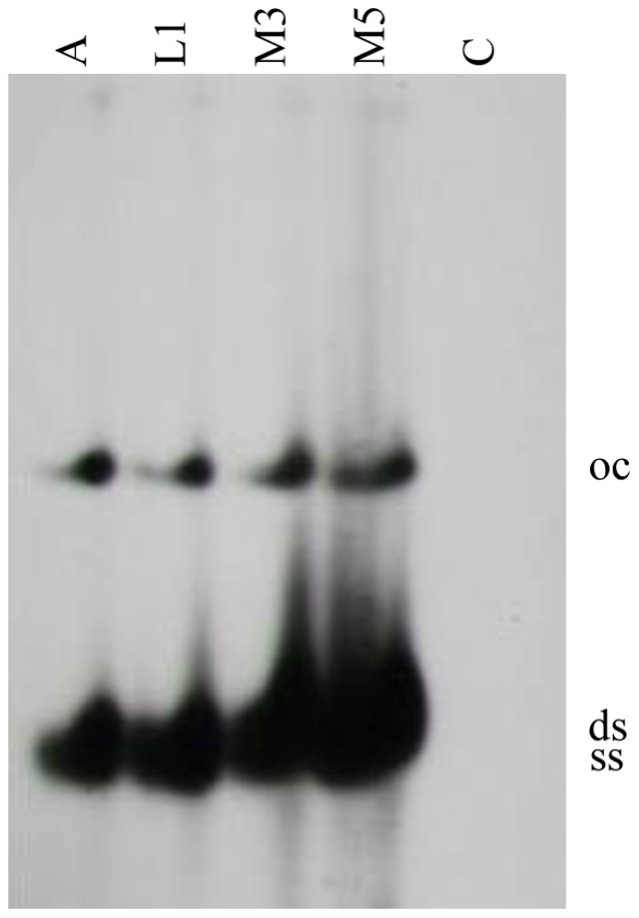
Southern blot analysis of TYLCSV DNA. DNA was extracted from the apex (A), the first leaf (L1) and the midrib of the third (M3) and the fifth (M5) leaves of infected tomato plants. Mock-inoculated tomato plants were used as negative control (C). A Rep gene specific probe was used for TYLCSV detection. Viral DNA forms are open circular double-stranded DNA (oc), covalently closed double-stranded DNA (ds) and single-stranded DNA (ss).

Prior to the microarray experiments, we verified that transcriptionally active double-stranded (replicative) viral forms were present in the selected material. DNA extracts from the apex, the first developing leaf and the midrib of the completely developed third and fifth leaves were examined by Southern blotting to determine which viral DNA forms were present: detection of both single- and double-stranded forms of viral DNA (Fig. 1) indicated that TYLCSV actively replicates in all the examined tissues.

### Identification of genes differentially expressed in TYLCSV-infected plants

RNA extracted from TYLCSV-infected and mock-inoculated plants was hybridized on the Agilent Tomato Gene Expression Microarray 4×44K. To reduce the biological variability, four biological replicates for each condition, each consisting of pools of four to five plants, were processed in parallel. Analysis of the Agilent One-Color RNA Spike-In Plot demonstrated similar and good performance of each single labelling and hybridization experiment, with consistency of data being confirmed by MA plots (data not shown).

Genes with a false discovery rate (FDR) <0.05 were considered differentially expressed. According to this criterion, we identified 3,604 genes differentially expressed (DE) between infected and mock-inoculated plants: 2,206 were up-regulated during viral infection and 1,398 were down-regulated (Table S2). The observed DE genes correspond to about 11% of the genes represented in the Agilent microarray platform used for the analysis. With its 33,484 genes (transcribed sequences), this microarray is representative of a relevant part of the tomato genome, consisting of 34,727 genes [21].

**Figure 2:**
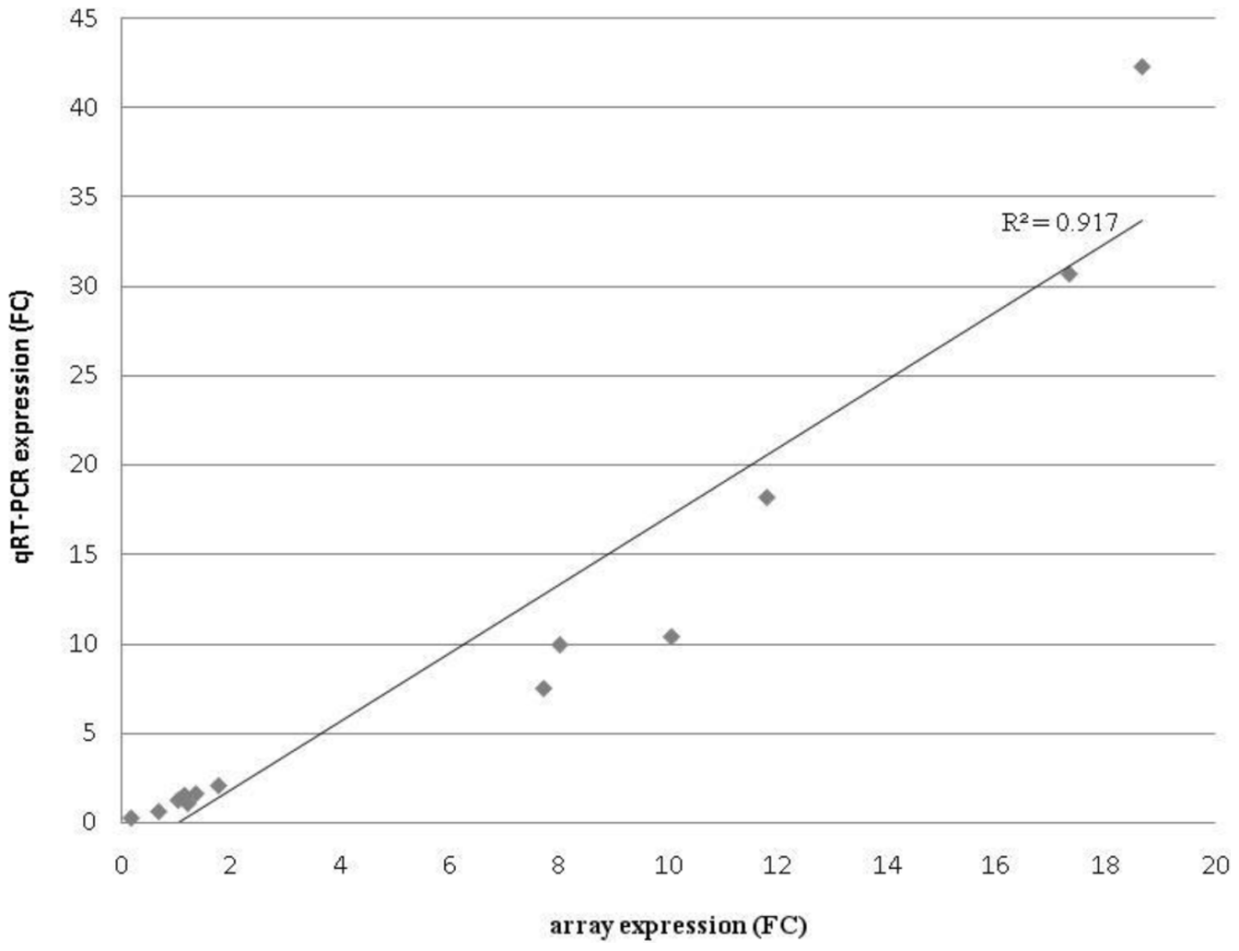
Correlation between microarray and qRT-PCR data. Expression values are expressed in fold changes (FC) in TYLCSV-infected plants in respect to mock inoculated ones

Microarray results were validated by quantitative real-time reverse-transcription polymerase chain reaction (qRT-PCR) using AK324262.1 (a gene coding for an ubiquitin-conjugating enzyme) as reference gene. This gene was not regulated by TYLCSV infection, as demonstrated by the not statistically significance of t-test analysis of Ct values (reported in Table S3). Differential expression of a subset of 14 DE genes was checked with the regulation observed in the microarray experiment being confirmed for all of them (Table 1); thus, a good correlation between microarray and qRT-PCR expression data was observed (R2 = 0.917; Fig.2). As already reported in different studies [13], [28], qRT-PCR fold changes tend to be greater than those measured by microarrays, particularly in the case of high fold change values. Raw qRT-PCR data can be found in Table S3.

**Table 1:** Validation of microarray data by qRT-PCR.

Gene id	Gene description	microarray	qRT-PCR
AK329513	hypothetical protein	18.67	42.35±6.11
AI777049	proline-rich protein	17.34	30.69±9.44
DB711969	nodulin-like protein	11.8	18.17±5.54
BW689844	zinc finger homeodomain protein 1	10.04	10.42±4.20
AI777966	hypothetical protein	8.02	9.94±1.88
AW442482	cysteine proteinase	7.72	7.46±2.11
BP895780	hypothetical protein	0.16	0.24±0.08
AJ243454.1	cyclin B1	1.15	1.49±0.19
AJ002590.1	cyclin D3	1.77	2.03±0.44
TC192990	DP1 transcription factor	1.03	1.22±0.11
BT012840.1	E2F transcription factor	1.35	1.55±0.16
TC216948	retinoblastoma-related protein	1.20	1.08±0.13
AJ515747.1	proliferating cell nuclear antigen	1.10	1.37±0.28
AJ441250	cyclin dependent kinase inhibitor	0.66	0.60±0.09

Expression values are expressed in fold change (FC) in TYLCSV-infected plants in respect to mock inoculated ones. qRT-PCR values are reported with the relative standard error.

### GO functional analysis of genes involved in TYLCSV infection

To characterize the function of genes differentially expressed in TYLCSV-infected plants, the array data were organized in functional categories according to Gene Ontology (GO) guidelines [29]. 33,484 genes (transcribed sequences) of the Agilent array were annotated with blast2go [25]. Considering the GO branch “biological process” as the most informative for functional characterization, 19,707 genes were annotated with at least one GO annotation and a total of 6,091 GO terms were involved (annotation percentage 59%). Among DE genes, 1,075 had at least one GO annotation in the “biological process” branch (annotation percentage 30%).

**Figure 3:**
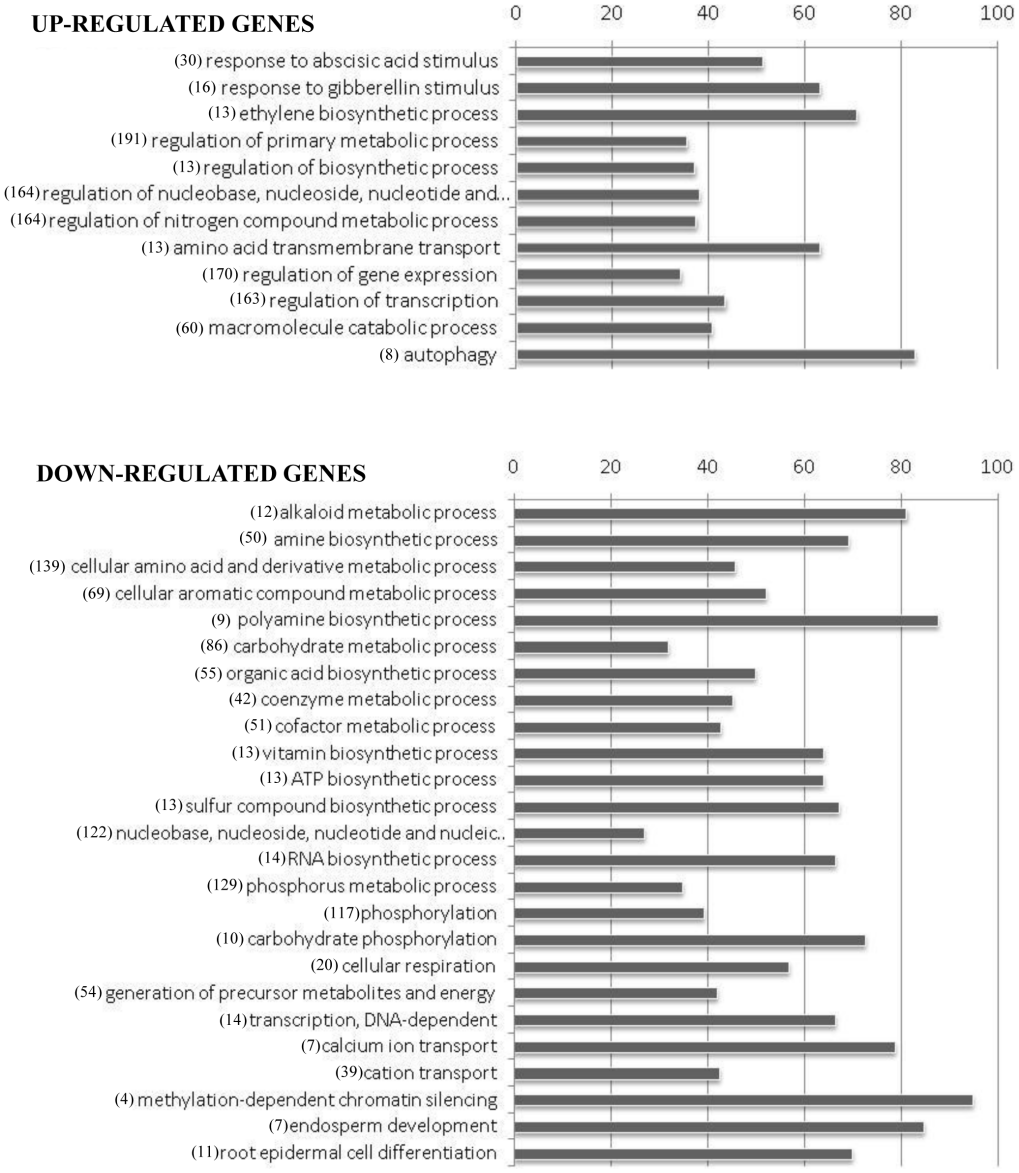
Over-represented GO categories (P<0.001) in genes differentially expressed in TYLCSV-infected plants in respect to mock-inoculated controls. Bars represent the percentage of regulated genes in addition to those expected by chance. GO categories were organized in five groups. Numbers of genes associated with each GO category are indicated in brackets.

DE genes were searched for overrepresented GO categories with a p-value <10^−3^ (see Table S4 for the complete list of overrepresented GO categories). To simplify the interpretation of the data, overrepresented categories were grouped and organized in two histograms (up-regulated and down-regulated genes) representing the percentage of regulated genes in addition to those expected by chance for each category (Fig.3).

Among the genes induced by TYLCSV infection, we observed a significant enrichment of those involved in hormone-related GO categories (*response to abscisic acid stimulus* (30 genes), *response to gibberellin stimulus* (16 genes), *ethylene biosynthetic process* (13 genes)) and those associated to the *nucleic acid* and *nitrogen compound metabolism* (164 genes). The same dataset was enriched in genes involved in *regulation of gene expression* (170 genes) and *transcription* (163 genes), including several MYB transcription factors and NAC domain proteins, and in the *macromolecule catabolic process* (60 genes), including several genes related to the ubiquitin proteasome pathway. The analysis of overrepresented functional categories also highlighted a significant enrichment, among the virus-induced DE genes, of those involved in *autophagy*: seven were up-regulated while only one was expected by chance. Five of them code for isoforms of the autophagy-related protein 8 (ATG8), one for an autophagy-related protein 9 (ATG9) and one for an autophagy-related protein 12 (ATG12).

The genes repressed during infection were significantly enriched in those involved in metabolic processes, suggesting a general reduction of primary and secondary metabolism. In particular, the overrepresentation of genes related to *generation of precursor metabolites and energy* (54 genes) among the down-regulated ones suggests a general repression of photosynthesis and respiration processes. Several kinases were present in the overrepresented functional category *phosphorylation* (117 genes), highlighting a negative impact of the infection on signalling. Moreover, we observed an enrichment in genes belonging to the GO category *transcription, DNA-dependent* (14 genes), mainly due to a repression of genes coding for RNA-polymerases II, which catalyzes synthesis of the precursors of mRNAs and most snRNAs and microRNAs. Finally, we found the functional category *methylation-dependent chromatin silencing* overrepresented among the down-regulated genes; two isoforms of the gene s-adenosyl-l-homocysteine hydrolase (SAHH), two adenosine kinases (ADK) and three s-adenosyl-l-methionine synthetases (SAMsynth) were repressed during viral infection.

### Identification of microRNA targets among the DE genes

MicroRNAs (miRNAs) are small endogenous RNAs involved in the regulation of several biological aspects as development [30], signal transduction and response to biotic and abiotic stresses [31], [32]. Recent studies have shown that begomovirus infection alters the expression of several miRNAs and postulated that this deregulation can be related to viral symptoms development [33], [34]. We searched our dataset of DE genes for targets of known tomato miRNAs (miRBase release 19, http://www.mirbase.org/), using the prediction tool psRNATarget (http://plantgrn.noble.org/psRNATarget/). A total of 29 DE genes were predicted targets of 11 tomato miRNAs (Table 2): four of them (miR156, miR159, miR171, miR172) were developmental miRNAs, conserved between plant families, and seven were family- (miR6022, miR6023, miR6024, miR6027, miR5303) or species-specific (miR1917, miR1918) miRNAs. A brief description of the target genes is presented hereafter.

**Table 2:** Differentially expressed transcripts targeted by tomato miRNAs.

Sly-miRNA	Target id	CS	TA	Target description		Target expression (log_2_FoldChange)
**conserved miRNAs**						
Sly-miR156	AK247958	1.5	10.60	squamosa promoter binding protein 6b	0.51	
	AK329145	3	17.58	unknown protein	−0.35	
sly-miR159	AF179247	3	13.53	1-aminocyclopropane-1-carboxylate synthase 8	−2.49	
Sly-miR171	AK319459	3	20.70	unknown protein	−0.43	
Sly-miR172	TC196115	2.5	18.33	receptor-like serine/threonine-protein kinase	1.68	
	EG553974	3	17.28	Inositol-tetrakisphosphate 1-kinase 1	1.09	
**Solanaceous-specific miRNAs**						
sly-miR6022	AF119040	1	21.86	Northern Lights Hcr9 gene cluster	1.39	
	BT013147	2.5	14.83	Hcr9	0.49	
	BT012922	3	10.80	homeobox-leucine zipper-like protein	0.80	
sly-miR6023	AF119040	1.5	19.19	Northern Lights Hcr9 gene cluster	1.39	
	BT013147	2	23.18	Hcr9	0.49	
	BM534947	3	22.39	cytochrome p450	0.72	
sly-miR6024	BT013733	2.5	17.35	atp binding	−0.87	
	AK322002	2.5	18.74	auxin-independent growth promoter protein	−0.36	
	AK326592	3	11.50	24 kda vacuolar protein	0.44	
	AI772612	3	9.16	pectinesterase	−2.02	
	TA40051_4081	3	13.48	chlorophyll a oxygenase	0.65	
	AK324689	3	16.20	chitinase A	0.77	
sly-miR6027	TC191372	2	16.36	tospovirus resistance protein Sw5	0.35	
sly-miR5303	AK319184	1	16.78	targeting protein for Xklp2 (TPX2) domain containing protein/Mpp10 domain containing protein	0.87	
	AW032180	2	19.42	serine/threonine protein kinase	0.48	
	AK320613	2	15.71	nac domain transcription factor	0.54	
	AI490899	2.5	14.20	phenylacetaldehyde reductase	−0.42	
	AK320244	2	15.00	unknown protein	0.81	
	DB716135	3	14.57	unknown protein	−0.82	
	AK324229	3	20.44	hydroxyphenylpyruvate reductase	1.06	
	AK325675	3	7.89	UBA/TS-N domain-containing protein, heat shock protein DnaJ	−0.77	
**Tomato-specific miRNAs**						
sly-miR1917	BT013773	2	10.19	SWI/SNF-related matrix-associated actin-dependent regulator of chromatin subfamily B member 1	−0.57	
sly-miR1918	TA55093_4081	0.5	17.79	fatty acyl-CoA synthetase domain containing protein	−0.93	
	BI926889	3	19.74	serine/threonine-protein phosphatase	−0.42	

Target prediction was obtained using psRNATarget. Expression values are reported as the log_2_(FoldChange) in TYLCSV-infected plants in respect to mock inoculated ones. CS: Complementary Score; TA: Target Accessibility.

Transcript AK247958, coding for a SQUAMOSA promoter binding protein 6b and target of the conserved Sly-miR156, was up-regulated (FC = 1.4) in infected tissues; searching among the tomato degradome sequences, available at the SoMART website [35], we identified 5′-uncapped remnants of polyadenylated mRNAs mapping at the predicted cleavage site, thus confirming the target prediction (data not shown). A second transcript coding for an unknown protein (AK329145) was also predicted to be a target of Sly-miR156, although with lower complementarity between the miRNA and the target sequence and lower target accessibility. This transcript was slightly down-regulated (FC = 0.78) in infected plants.

Transcript AF179247 coding for the 1-aminocyclopropane-1-carboxylate synthase 8 (ACS8) that catalyzes the rate-limiting step in the ethylene biosynthetic pathway in plants, was down-regulated (FC = 0.18) in TYLCSV-infected plants. Its mRNA is targeted by Sly-miR159, one of the most conserved miRNA in land plants [36], which also targets several MYB transcription factors involved in flowering and male fertility [37]. However, a novel target not related to MYB transcription factors has been recently discovered [38].

Transcript AK319459 coding for an unknown protein repressed (FC = 0.74) in infected plants is targeted by Sly-miR171, known for its role in controlling the transitions from juvenile to adult, and from adult to reproductive phases [39].

Two transcripts (TC196115 and EG553974) coding for a receptor-like serine/threonine-protein kinase and an inositol-tetrakisphosphate 1-kinase 1, both induced by infection (FC = 3.21 and FC = 2.13 respectively), are targets of Sly-miR172, known to mediate the control of the flowering process [40].

Sly-miR6022, Sly-miR6023, Sly-miR6024 and Sly-miR6027 belong to miRNA families up to date identified only in solanaceous species, and able to target resistance (R) genes [41]. The above mentioned miRNAs target several DE genes regulated by TYLCSV infection. Three mRNA transcripts are targets of miR6022: two of them (AF119040 and BT013147), experimentally confirmed by 5′-RACE [41], code for resistance proteins belonging to the Hcr9 (homolog of *Cladosporiumfulvum* resistance gene *Cf-9*) gene family, and one (BT012922) codes for an Homeobox-leucine zipper-like protein. All were up-regulated in infected plants (FC = 2.63, 1.40 and 1.74 respectively). Sly-miR6023 targets three transcripts: two, coding for Hcr9 proteins, in common with miR6022 (i.e. AF119040 and BT013147), and one (BM534947), also up-regulated (FC = 1.65) in infected tomato plants, encoding a cytochrome p450. Sly-miR6024 targets six different transcripts: three (a 24 kDa vacuolar protein, a chlorophyll a oxygenase, and a chitinase A) were up-regulated (FC =  1.35, 1.57 and 1.70 respectively) in infected plants and three (an ATP binding protein, an auxin-independent growth promoter protein and a pectinesterase) were down-regulated (FC = 0.55, 0.77 and 0.25 respectively). Sly-miR6027 targets a transcript (TA56186_4081/TC191372) coding for a tospovirus resistance protein *Sw5*, up-regulated (FC = 1.28) in TYLCSV-infected tomato plants. Searching the available tomato degradome libraries using SoMART [35], we found 5′-uncapped remnants mapping on this transcript, thus confirming the cleavage at the predicted position.

Sly-miR5303, up to now identified only in tomato and tobacco, is predicted to target eight transcripts: five (a targeting protein for Xklp2 (TPX2) domain containing protein/Mpp10 domain containing protein, a serine/threonine protein kinase, a NAC domain transcription factor, an unknown protein and an hydroxyphenylpyruvate reductase) were induced (FC = 1.83, 1.40, 1.46, 1.75 and 2.09 respectively) by viral infection, and three (a phenylacetaldehyde reductase, an unknown protein and a UBA/TS-N domain-containing protein/heat shock protein DnaJ) were repressed (FC = 0.75, 0.57 and 0.59 respectively). The transcript with the best alignment score, associated with a good level of target accessibility, was AK319184, coding for the TPX2/Mpp10 domain containing protein.

Finally, among our DE genes, we identified few targets of tomato-specific miRNAs. The transcript BT013773 coding for a SWI/SNF-related matrix-associated actin-dependent regulator of chromatin subfamily B member 1 is a target of Sly-miR1917 and was repressed (FC = 0.67) by TYLCSV infection. Two transcripts (TA55093_4081 and BI926889), both repressed in infected tissues (FC = 0.53 and FC = 0.74 respectively), are targeted by Sly-miR1918. The first one, coding for a fatty acyl-CoA synthetase domain containing protein, was validated by Moxon and co-workers using the 5′-RACE technique [42]. The second one, coding for a serine/threonine-protein phosphatase, shows a lower level of complementarity with the miRNA sequence and a lower target accessibility, and remains to be validated.

### Comparison with other geminiviral transcriptomic studies

Transcriptomic data on geminivirus-infected plants are very limited and, up to now, the only two microarray studies dealing with the impact of geminivirus infection on host gene expression focused on the model plant *Arabidopsis thaliana* infected by either *Cabbage leaf curl virus* (CabLCV) [19] or *South Africa cassava mosaic virus* (SACMV) [20], both bipartite begomoviruses. In order to compare our expression data with those available from the literature, tomato transcripts used by Agilent to design microarray probes were mapped, using blastn, on SGN-unigene tomato transcripts available in the Solanaceae Genomics Network database (http://solgenomics.net/), and only hits with e-value equal to 0.0 were retained for further analysis. Out of 3,604 tomato DE transcripts, 3,250 (90% of tomato DE transcripts) had a correspondent SGN-unigene sequence. According to the mapping available at the Solanaceae Genomic Network database, 2,411 of them (67% of tomato DE transcripts) had a homologue in Arabidopsis.

**Figure 4:**
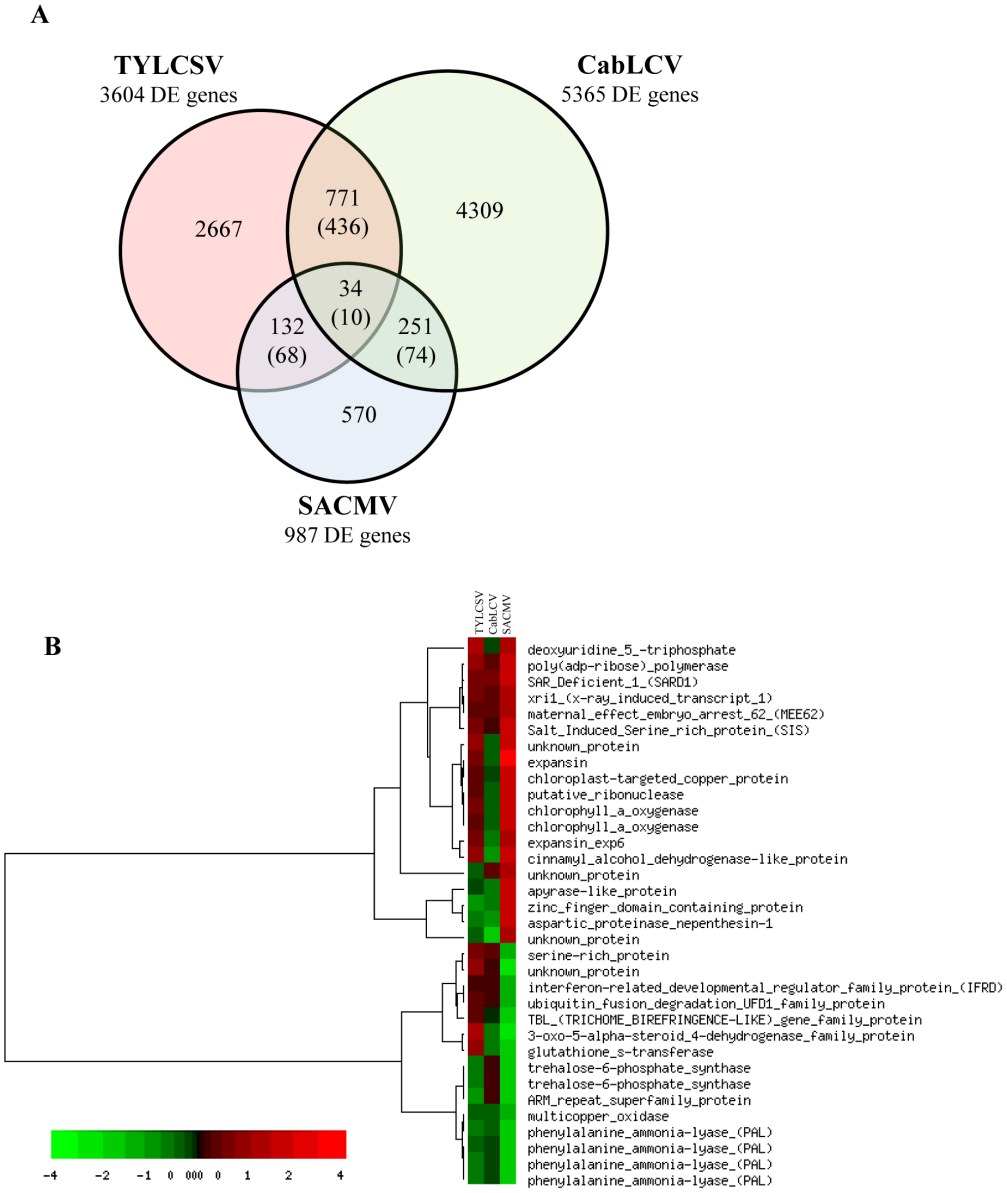
Comparison of the genes differentially expressed in TYLCSV-tomato, CabLCV-Arabidopsis and SACMV-Arabidopsis (36 dpi). (A) Venn diagram. Values in brackets indicates the number of genes regulated in the same direction. (B) Hierarchical clustering.

As a first step, we compared our data with those obtained on Arabidopsis infected by CabLCV [19], where experiments were performed using well developed fully symptomatic leaves, as in our case. Comparing the genes differentially expressed in response to TYLCSV and CabLCV infections, we found that 805 DE genes were in common: 446 showed similar expression trends (297 genes up-regulated and 149 down-regulated) and 359 showed opposite trends (Fig 4A and Table S5). To highlight biological processes important for both infections, we proceeded to a functional characterization of these commonly regulated DE genes, and, using the bioinformatic tool Mapman (http://mapman.gabipd.org), we identified the overrepresented functional categories. Several genes involved in response to biotic stress, (i.e. PR proteins, heat shock proteins and glutatione-S-transferases) as well as genes involved in ethylene metabolism (mainly coding for 1-aminocyclopropane-1-carboxylate oxidase homologs (ACCO)/E8 proteins) were induced by both TYLCSV and CabLCV. Moreover, these geminiviruses commonly up-regulated a significant number of genes related to the protein degradation pathway, in particular proteases (mainly cysteine proteases), and proteins related to the ubiquitination pathway and several autophagy-related proteins (different ATG8 isoforms).

Focusing on genes repressed by both TYLCSV and CabLCV, the functional analysis highlighted that several of them were involved in RNA transcription (mostly RNA polymerases II). Genes coding for proteins involved in the amino acid metabolism, nucleotide metabolism and response to abiotic stress were also commonly repressed.

Pierce and Rey [20] sampled the rosette leaves closest to the meristem tip and investigated the transcriptional response of Arabidopsis to SACMV infection at 14 dpi (initial symptoms), 24 dpi (fully symptomatic) and 36 dpi (well established late infection). Comparing our list of DE genes with those obtained for SACMV/Arabidopsis interaction, we found that the number of common DE genes was lower at 14 dpi (42 genes differentially expressed in both infections), intermediate at 24 dpi (103 DE genes in both infections) and higher at 36 dpi, with 166 genes differentially expressed in response to both viruses (78 of them were regulated in the same direction) (Fig.4A and Table S5). Since our expression data were referred to plants with a well-established TYLCSV infection, we focused our comparison on the transcriptional responses of Arabidopsis to SACMV infection at 36 dpi, when, as expected, the number of common DE genes is the highest. However, the analysis of overrepresented functional categories among the genes regulated in the same direction by TYLCSV and SACMV did not yield significant results, probably due to the different biological material considered: the midrib of fully developed leaves for tomato/TYLCSV and young developing leaves for Arabidopisis/SACMV. It is worth mentioning that, among the 88 genes regulated in the opposite way during the two infections, we observed genes involved in the ethylene biosynthesis, particularly 1-aminocyclopropane-1-carboxylate oxidases, which were induced by TYLCSV but repressed by SACMV.

Comparing data on TYLCSV-infected tomato plants with both those on CabLCV-infected Arabidopsis plants and SACMV-infected Arabidopsis plants, we observed that only 34 genes were present in all three datasets (Fig.4A and B). Out of them, five were induced by all the three viruses and five were repressed (four of which were phenylalanine ammonia-lyases (PAL)).

## Discussion

### Hormone-mediated responses

Gibberellins (GAs) are a class of tetracyclic diterpenoid phytohormones that play essential roles in regulating many aspects of the plant development. GA promotes plant growth by stimulating degradation of negative growth regulators called DELLA proteins, but, emerging evidence suggest that GA signalling components play also major roles in plant disease resistance and susceptibility and that, in particular, viral proteins can affect GA signalling components in plants [43]. *Rice dwarf virus* (RDV) infection represses the rice ent-kaurene oxidases, mediating the GA biosynthetic pathway, causing a significant reduction of GA level. Infection of rice plants with RDV results in stunting and darkening of leaves, symptoms characteristic of GA-deficient rice mutants; treatment of infected plants with GA is able to restore normal growth phenotype [44].

In our microarray data, the analysis of functional categories highlighted an overrepresentation of genes belonging to the GO category *response to gibberellin stimulus* (16 genes). Following this observation we were prompted to search for DE genes involved in GA metabolism and signalling. Two out of three genes coding for gibberellin-20-oxidases, involved in the biosynthesis of GAs, were induced (FC = 2,6 and FC = 1,6). On the other hand, four genes coding for gibberellin-2-oxidases, which inactivate GAs by introducing a hydroxyl at the 2β position [45], were all up-regulated (FC = 3.5, 3.3, 1.8 and 1.7). In rice, under low GA concentrations, the DELLA transcription factor SLR1 represses the GA responses. When GA level increases, the soluble receptor GID1 binds GA, forming the GID1-GA complex. This interacts with SLR1 and leads to its degradation by the 26S proteasome, thus releasing the repressive state of GA responses [46]. In our data, we found that two genes coding for the nuclear gibberellin receptor GID1 were induced in TYLCSV infected plants (FC = 2.1, 1.6), and a transcript coding for the GAI protein, a repressor of GA signalling similar to the rice protein SLR1 [47], was repressed (FC = 0.4). All the above observations suggest a role for GA in plant-TYLCSV interaction, with an increase in GA levels during infection, partially counteracted by the induction of the GA-inactivating enzymes gibberellin-2-oxidases.

Recent studies revealed a complex crosstalk between GA, ABA and JA signalling mediating plant growth and response to abiotic and biotic stresses [48], [49]. We found that the GO category *response to abscisic acid stimulus* was overrepresented among up-regulated genes, and the gene coding for the abscisic acid 8'-hydroxylase, a key enzyme for ABA degradation [50], was down-regulated (FC = 0.4). These data suggest an increase of ABA level during TYLCSV infection. Actually, in addition to playing a key role in developmental processes and in response to abiotic stresses, ABA has been shown to be involved in the interaction between RNA viruses and their host plants. ABA level was observed to increase in tomato shoots infected by *Tomato spotted wilt virus* [13], [51] and in tobacco leaves systemically infected by TMV [52]; interestingly, treatment with exogenous ABA improved resistance to TMV infection in tobacco [53]. A first relation between ABA and geminivirus infection was observed in Arabidopsis plants inoculated with the curtovirus, *Beet severe curly top virus* (BSCTV). ATHB12 and ATHB7 genes, coding for two transcription factors belonging to the homeodomain-leucine zipper family, previously shown to be induced by abscisic acid, were also induced in symptomatic BSCTV-infected tissues. Moreover, ATHB12 expression was correlated with several morphological abnormalities such as leaf curling, stunting, and callus-like structures in infected Arabidopsis plants [54]. As far as we know, no other evidence of the ABA involvement in geminivirus infection was found so far.

JA has recently received attention in geminivirus research [11]. Although we do not see overrepresentation of genes involved in JA metabolism or in JA response, changes in expression of some individual JA-related transcripts were observed.

Coronatine-insensitive 1 (COI1), a key regulatory protein in the JA-signalling pathway, was up-regulated (FC = 1.4) in TYLCSV infected tomato plants, but a transcript annotated as jasmonate insensitive 1 (JAI1), a nuclear-localized leucine zipper transcription factor induced by JA in a COI1–dependent manner [55], was down-regulated (FC = 0.6). The equilibrium between JA and MeJA also plays a key role in regulating JA-mediated responses in plants. Among our DE genes, we found that a transcript coding for an S-adenosyl-L-methionine:jasmonic acid carboxyl methyltransferase (JMT), catalyzing the synthesis of MeJA from JA [56], was repressed (FC = 0.41) in TYLCSV-infected plants, and two transcripts coding for two methyl jasmonate esterases (MJE), producing JA from MeJA [56], were induced (FC = 1.59 and  = 1.69). Taken together our data suggest that a decrease of MeJA level (possibly active in plant-defence responses) occurs in favour of JA level and highlight the complexity of JA-based responses of plants to geminiviral infection.

The level of JA can influence the relationship between the host plant and the viral insect vector *Bemisia tabaci*. Actually, it has been observed that tomato mutants with impaired JA production, negatively affecting the levels of constitutively emitted volatile organic compounds (i.e. terpenoids), are preferentially selected by insect females for ovideposition [57]. In this context is intriguing to speculate that the changes in JA level due to TYLCSV infection may improve the viral fitness, favouring the vector/host plant relationship.

Salicylic acid (SA) is another hormone with an important role in many plant-pathogen interactions and it is known to activate local and systemic defence responses, particularly against biotrophs [58]. Activation of the SA pathway is a typical plant response to RNA viruses [59] and a similar induction of SA-mediated responses was observed in the Arabidopsis-CabLCV infection [19]. Surprisingly, we found nine transcripts, coding for phenylalanine ammonia lyases, key enzymes in the SA biosynthesis [60], all down-regulated (FC ranging from 0.68 to 0.55) in TYLCSV-infected tissues, thus suggesting a decrease in SA levels in infected plants. Moreover, only two genes coding for proteins belonging to the SA-mediated signalling pathway were regulated during infection, one induced (ALD1, FC = 2.1) and the other repressed in infected tissues (SAG101, FC = 0.7). The differences observed between our data and those in literature could suggest a different role of salicylic acid in TYLCSV infection but they could also be explained considering that, instead of the whole leaf, only portions of the midrib from mature leaves were used in our analysis.

**Figure 5:**
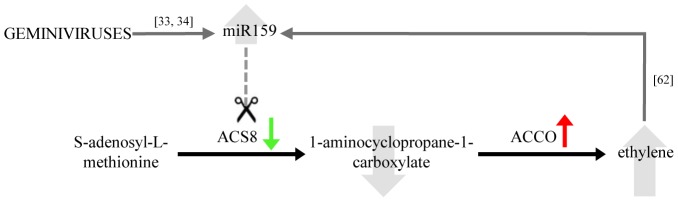
Hypothetical role of miR159 in ethylene regulation during geminivirus infection. Green and red arrows indicate the regulation of genes during TYLCSV infection according to microarray data. Numbers indicate literature references, and dashed line prediction of miR159 targeting of 1-aminocyclopropane-1-carboxylate synthase 8 (ACS8). ACCO: 1-aminocyclopropane-1-carboxylate oxidase.

Ethylene (ET) is a plant hormone involved in the regulation of plant defences [43]. Both TYLCSV and CabLCV affect ET metabolism, mainly activating the 1-aminocyclopropane-1-carboxylate oxidase (ACCO), which regulates the final step of ET biosynthesis [61]. This enzyme is also induced by ToLCNDV [17] and PepGMV [18]. On the other hand, TYLCSV down-regulated (FC = 0.18) the transcript coding for the 1-aminocyclopropane-1-carboxylate synthase 8 (ACS8), which catalyses the rate-limiting step in the ethylene biosynthetic pathway [61], while no differential expression of this gene was observed in Arabidopsis infected by CabLCV or by SACMV. Searching for targets of known miRNAs among genes regulated during infection, we found that ACS8 transcript is a predicted target of miR159 (Table 2). Even if most of the known miR159-targets are MYB transcription factors, a novel target not related to MYB genes has been recently discovered [38], and a relationship between miR159 and ethylene metabolism has been already suggested in a study focused on tomato fruit ripening [62]. Actually, miR159 is responsive to ET, being down-regulated by application of the exogenous ethylene precursor ACC in *Medicago truncatula* roots [63] but induced in tomato fruits by treatment with exogenous ET [62]. On the other hand, a relation between miR159 and geminivirus infection was noticed in *N. benthamiana* infected by TYLCV [33], as well as in tomato and chilli plants infected by ToLCNDV, and in ToLCV-infected tomato where in the level of miR159 increased as infection progressed [34]. Based on these data we suggest that, during TYLCSV systemic infection, a complex tuning of ethylene metabolism involving miR159 could take place (Fig.5). According to our hypothesis, geminivirus infection would induce the miR159 expression that drives the cleavage of the ACS8 transcript, thus reducing the ACC level and supporting the miR159 induction. At the same time, ACCO enzyme would be up-regulated, probably to compensate ACC level reduction, and the consequent decreasing of ET level, suggested by the down-regulation of the ET-responsive genes ERF2 (FC = 0.4), ERF4 (FC = 0.3), and ETR4 (FC = 0.7). It is worth remembering that we are looking at a fully established infection, where a dynamic equilibrium between virus attacks and host defences is expected to take place.

### Autophagy and infection

Autophagy is an evolutionarily conserved mechanism for intracellular recycling whereby large protein complexes and aggregates, organelles, and even invading pathogens are encapsulated in specific vesicles called autophagosomes, which are then engulfed and digested by vacuoles/lysosomes. This process is essential for tissue homeostasis and development, and plays a critical role in the ability of plants to survive nutrient starvation as well as to exposure to abiotic and biotic stresses [64].

The analysis of overrepresented functional categories highlighted a significant enrichment of autophagy-related transcripts among the genes up-regulated during the TYLCSV/tomato compatible interaction. All these transcripts were related to the formation of autophagosomes. In particular, several transcripts coding for isoforms of ATG8, key regulator and marker of the autophagic process, were induced during infection. ATG8 is an ubiquitin-like protein required for the formation of autophagosomal membranes. During the autophagic process, ATG8 anchors to the autophagosomes membranes by a carboxyl terminal phosphatidylethanolamine lipid (PE). Interestingly, activation of ATG8 genes was also observed in Arabidopsis infected by CabLCV [19]. Other TYLCSV-induced transcripts coded for ATG9, involved in the membrane delivery to autophagosomes, and ATG12, an ubiquitin-like protein involved in the formation of the ATG8-PE complex [64].

Being intracellular parasites, viruses deal with the autophagic machinery during the infection process. In this respect, autophagy can play both anti-viral and pro-viral roles in viral life cycle and pathogenesis. On one hand, autophagy proteins can target viral components for lysosomal degradation (i.e. xenophagy) and play a role in initiating immune response to infection [64], [65]. In this context, we can suppose that the observed up-regulation of autophagy genes is a host attempt to counteract viral invasion by elimination of exogenous viral particles.

On the other hand, some animal viruses are able to hijack autophagy to foster their intracellular growth (reviewed in [66]). In plants it is known that virus multiplication sequesters important cellular components, causing cell starvation and activation of genes involved in programmed cell death (PCD) [19]. Cell death would be detrimental to viruses, in particular those like TYLCSV, which are restricted to a few cells in the phloem. Activation of autophagy by negatively regulating PCD [67], would prevent cells to die, thus maintaining a favourable environment for the virus.

### Fine tuning of plant innate immune response

Plant defence responses against pathogen attacks are mediated by resistance (R) genes coding for leucine-rich repeat (LRR) and intracellular nucleotide binding (NB)-LRR proteins able to directly or indirectly recognize pathogen effectors and trigger host immunity responses [68]. It has been recently observed that several viral proteins can function as effector proteins, triggering R-gene mediated host responses [69]. Moreover, different studies revealed the existence of regulatory cascades affecting a large portion of NB-LRR resistance genes and initiated by specific miRNAs (reviewed in [70]). Li and co-workers [41] described two tobacco miRNAs (i.e. Nta-miR6019 and Nta-miR6020) able to guide a sequence-specific cleavage of TIR-NB-LRR immune receptor N transcript, which confers resistance to *Tobacco mosaic virus* (TMV). Furthermore, they identified in three solanaceous species (tobacco, tomato and potato), 21-nt and 22-nt miRNA families, whose members direct cleavage of transcripts coding for R genes, triggering the production of secondary small-interfering RNAs, and demonstrated that the role of these miRNAs in regulating LRR and NB-LRR immune receptors is conserved in Solanaceae.

In our work, we identified eleven target transcripts for Sly-miR6022, -miR6023, -miR6024 and -miR6027, all belonging to R genes-targeting miRNAs families (Table 2). Most of them, particularly those already validated and coding for resistance proteins (two *Hcr9* resistance proteins and one *Sw5* resistance protein), were up-regulated by viral infection, thus suggesting that the targeting miRNAs involved in innate immune receptor gene regulation and pathogen resistance in Solanaceae, are repressed during TYLCSV infection. Moreover, as already observed [41], both Sly-miR6022 and -miR6023 target the same *Hcr9* transcripts, possibly indicating that tuning of these R genes is important for TYLCSV infection. *Hcr9s* are a large family of resistance genes, homologous to the *Cf9* gene, that confer resistance against *Cladosporium fulvum* through recognition of different pathogen-encoded avirulence determinants [71]. A tomato mutant expressing a recombinant Hcr9 autoactive protein showed stunting, a symptom of TYLCSV infection, and constitutive expression of defence genes PR1 and PR5 [72]. Interestingly, transcripts coding for these two proteins were also up-regulated in our data. Sly-miR6027, as supported by the identification of the predicted RNA cleavage products (data not shown) among the tomato degradome sequences available at the SoMART website [35], target a transcript coding for the tospovirus resistance protein *Sw5*. The tomato gene *Sw5* belongs to the coiled-coil NB-LRR class of plant resistance genes and confers resistance to *Tomato spotted wilt virus* through a ‘gene-for-gene’ recognition of pathogen avirulence factors. *Sw5* belongs to a multigene family whose members are dispersed throughout the tomato genome and may confer resistance to a variety of pathogens [73]. Its role in geminivirus infection remains to be elucidated.

These data suggest the involvement of plant innate immunity defence mechanisms in plant/geminivirus interaction and indicate that TYLCSV can directly/indirectly intervene on the regulation of specific R genes in order to establish a successful infection.

### Shared geminiviruses-activated pathways

The clustering analysis of the expression profiles highlighted a higher level of similarity between CabLCV [19] and SACMV [20] datasets than between each of them and TYLCSV (data not shown). This was probably due to the different viruses and plants considered. In fact, TYLCSV is a strictly phloem-limited monopartite begomovirus, while CabLCV and SACMV do not have that restriction and their genome is bipartite. Moreover, the differences in expression patterns may reflect different levels of adaptation: TYLCSV/tomato is a natural system, while the other viruses infect Arabidopsis only in laboratory conditions.

A deeper comparison of our data with those obtained for CabLCV/Arabidopsis interaction indicated that about half of the genes differentially expressed in response to both TYLCSV and CabLCV were regulated in the same direction. Both geminiviruses activate a generic response to biotic stress, involving PR proteins, heat shock proteins and glutatione-S transferases. This host reaction was also highlighted using the mRNA-seq approach in pepper plants infected by the PepGMV [18] and, by subtractive hybridization, in tomato plants infected by ToLCNDV [17]. Similar plant response to biotic stresses was observed in the majority of RNA virus infections [13], [74].

The analysis of functional categories significantly represented among genes repressed by both TYLCSV and CabLCV highlighted a general repression of amino acid metabolism and in particular of enzymes belonging to the methionine cycle (also known as Yang cycle). Actually, two genes coding for S-adenosylmethionine synthetase, two coding for S-adenosyl-L-homocysteine hydrolase, one coding for a homocysteine methyltransferase, and one coding for an adenosine kinase (ADK) were down-regulated in both cases. Geminiviruses are DNA viruses that replicate in the nucleus of host cells, associate with cellular histone proteins and form viral minichromosomes [75], and are subjected to transcriptional gene silencing through methylation. It has been observed that *in vitro* methylation of geminiviral DNA greatly reduces the virus ability to replicate in plant protoplasts [76], [77] and that C2 and L2 geminiviral proteins can interact and inhibit ADK [78], [79], an enzyme required for efficient production of the methyl group donor S-adenosyl methionine and essential for the methylation process [80]. Moreover, Raja and co-workers [75] highlighted the importance of methylation in plant-geminivirus interaction showing that methylation-deficient plants become highly susceptible to geminivirus infection and develop more severe symptoms than wild-type plants. Therefore, chromatin methylation was proposed as a plant defence mechanism against DNA viruses. In this context, the repression of Yang cycle can be regarded as an attempt performed by geminiviruses to inhibit global methylation and counteract host defences. Whether repression of enzymes belonging to the methyl cycle is a consequence of the ADK inhibition or other regulatory processes are involved, remains to be elucidated.

Functional analysis of overrepresented GO categories among DE genes in TYLCSV-infected tomato also showed a significant enrichment of genes involved in the macromolecule catabolic process. Further analyses highlighted that the induction of the protein degradation pathway, and in particular the ubiquitin-dependent degradation pathways, is shared in both TYLCSV and CabLCV infections. Ubiquitination is a post-translational modification consisting in the attachment of ubiquitin (Ub) to cellular proteins, leading to their degradation by the ubiquitin proteasome system (UPS) or, alternatively to other cellular processes such as subcellular localization, protein activation or protein-protein interaction; it is considered an important regulatory mechanism in plants [81], [82]. This process has a relevant role in regulation of plant-pathogen interactions (reviewed by [83]) and several studies highlighted the mechanisms evolved by viruses to hijack the UPS (reviewed by [84]). We found that several genes belonging to the ubiquitination pathway were induced by both TYLCSV and CabLCV. Interestingly several cysteine proteases, able to reverse ubiquitination [84] were also commonly induced, suggesting that the fine-tuning of the ubiquitination process can be relevant in regulating the complex interaction between the virus and its host plant.

Several genes coding for RNA polymerase II (Pol II) were also repressed by both TYLCSV and CabLCV. This polymerase is involved in the synthesis of both protein-coding and non-coding RNAs such as small nuclear, nucleolar and microRNAs, but also in the synthesis of viral transcripts. In the dynamic equilibrium between the host and the pathogen, leading to the development of systemic infection, it is difficult to explain the observed down-regulation of Pol II coding genes. We speculate that it might represent the plant attempt to counteract viral infection by limiting the production of viral transcripts; however, the observed repression could be also associated to a decreased concentration of plant proteins involved in the antiviral response.

Geminiviruses are known to activate DNA replication and core cell cycle genes in fully differentiated infected cells, thus activating the host DNA synthesis machinery needed for viral DNA replication [85]. Contrarily to what observed for CabLCV [19] and SACMV [20], we could not detect in our dataset any indication of cell cycle activation probably because of the very low number of infected cells. The inability of the approach to highlight such change underscores the difficulty to investigate cell-autonomous processes, particularly for phloem-limited viruses, and the need to study infected cells instead of cell tissues.

## Conclusions

Virus-induced gene expression has been extensively studied in several compatible interactions between RNA viruses and their host plants. On the other hand, our knowledge on transcriptional responses of host plants to geminivirus infection has been limited, up to now, by the paucity of well-characterized host model systems suitable for global transcriptomic analysis, and by the technical limitations due to the small fraction of host cells typically infected by these DNA viruses.

In this study we presented the first global microarray analysis performed on a monopartite phloem-limited geminivirus infecting its natural host. Our results highlighted a series of responses so far observed only in the TYLCSV-tomato compatible interaction, such as the induction of GA- and ABA-responsive genes, the activation of the autophagic process and the fine tuning of the plant immune system. On the other hand, comparison with previous studies showed the existence of responses common to other geminivirus infections, such as the induction of the ubiquitination pathway and the inhibition of the methyl cycle.

Our results provide new insight into the biology of geminivirus–plant interactions and represent a step toward the identification of host genes required for successful virus infection and the consequent set up of efficient control strategies.

## 

## Supporting Information

Table S1 **List of primers used in quantitative Real-Time PCR.**
(DOC)

Table S2 **List of DE genes in TYLCSV-infected tomato.**
(XLS)

Table S3 **Quantitative Real-Time PCR data.**
(XLS)

Table S4 **Overrepresented GO categories among DE genes in TYLCSV-infected tomato.**
(XLS)

Table S5 **Comparison of datasets of DE genes in TYLCSV-infected tomato with CabLCV- and SACMV-infected Arabidopsis.**
(XLS)
