# Supplementary material for: Transcriptomics of the Interaction between the Monopartite Phloem-Limited Geminivirus Tomato Yellow Leaf Curl Sardinia Virus and Solanum lycopersicum Highlights a Role for Plant Hormones, Autophagy and Plant Immune System Fine Tuning during Infection
Source: PLoS One. 2014 Feb 28;9(2):e89951. doi: 10.1371/journal.pone.0089951 (PMC3938563; doi:10.1371/journal.pone.0089951)
Supplement: Table S1 — List of primers used in quantitative Real-Time PCR. (DOC) [file pone.0089951.s001.doc]

Supplementary Table S1 – List of primers used in quantitative Real-Time PCR

| **Gene** | **Primer forward** | **Primer reverse** |
| --- | --- | --- |
| AK324262 | GGAGTACTTTGGAAGCAAATGG | GGAGTACTTTGGAAGCAAATGG |
| AK329513 | ACTTTGATGCAGCAGGTAGACA | GAAAGGAGGTTTGGATAGAGCA |
| AI777049 | TAATAGTCGGGTCTCCACCAAC | GGAAATTAATGAGGCAGAGTGG |
| DB711969 | TTAGTGCTGTTTCTTGGGGAAT | ATGCTATGGCTCAAAGACCAAT |
| BW689844 | TGTTGAGTGCCAGAGAAATCAT | AAGCGGAAGTACATTCAGAAGC |
| AI777966 | ACATTTGGCATGATAGCAACAG | CACAAATGCAGCAACAAGAAAT |
| AW442482 | AGTGACAGCACCTTTCTTCCTC | TGCAGACATGACTAACCATGAA |
| BP895780 | GATTTGCATGGAGGTTGGATAC | TCGTCTGCATGTCCTCTATACG |
| AJ243454 | CACACAAAAGGTTTGAGCTGAG | AGCATTGAGCTGATGCAAAGTA |
| AJ002590 | TGAATCCAGTAACCCCATTTTC | GGTACTTTTCTCCAATGCAAGG |
| TC192990 | TGGGAAAAAGAAAAGAGCTCAG | TCGTAGACTCGTCGTCTGATGT |
| BT012840 | CAATTCCAACATTGGCTACTGA | CTGTCCTCCACATGTCAGTGAT |
| TC216948 | CAGGAGCTGTTTTTGTCAAGTG | GGGGAGTCATTGATGTTGAAAT |
| AJ515747 | AAGGATCTGGTGAACGATGC | CAGCCTTGATGGTGATGATG |
| AJ441250 | AGGCATAAATTGTGGATTAGGC | GGAAGTAAAGGGCCAGATTTTT |
